# Polycythemia among Patients with Chronic Obstructive Pulmonary Disease Admitted to the Department of Medicine in a Tertiary Care Center: A Descriptive Cross-sectional Study

**DOI:** 10.31729/jnma.8125

**Published:** 2023-04-30

**Authors:** Krishna Bahadur Thapa, Ashok Paudel, Sujan Dhital, Abhash Shrestha, Liladhar Ojha, Akarsha Shrestha

**Affiliations:** 1 Department of Medicine, Gandaki Medical College, Ritthepani, Kaski, Pokhara, Nepal; 2 Department of Medicine, Kathmandu Medical College, Sinamangal, Kathmandu, Nepal

**Keywords:** *chronic obstructive pulmonary disease*, *polycythemia*, *prevalence*

## Abstract

**Introduction:**

Chronic obstructive pulmonary disease is a preventable and treatable disease marked by persistent airflow limitation. Abnormal rise of haemoglobin and/or hematocrit in peripheral blood is known as polycythemia which includes increased haemoglobin: greater than 16.5 g/dl in men or greater than 16.0 g/dl in women and increased hematocrit: >49% for men and >48% for women. Men, current smoking, impaired carbon monoxide diffusing capacity, severe hypoxemia, and high altitude living are risk factors associated with an increased risk for secondary polycythemia. Polycythemia contributes to the development of cor-pulmonale and pulmonary hypertension, which are linked to poor prognosis. This study aimed to find out the prevalence of polycythemia among patients with chronic obstructive pulmonary disease admitted to the department of medicine in a tertiary care centre.

**Methods:**

A descriptive cross-sectional study was conducted among patients with chronic obstructive pulmonary disease admitted to the Department of Medicine in a tertiary care centre after receiving ethical approval from Institutional Review Committee (Reference number: 153/079/080). The study was conducted from 15 September 2022 to 2 December 2022. Data were collected from the hospital records. A convenience sampling method was used. Point estimate and 95% Confidence Interval were calculated.

**Results:**

Among 185 patients, Polycythemia was seen in 8 (4.32%) (1.39-7.25, 95% Confidence Interval) patients among which 7 (87.5%) were females and 1 (12.5%) were male.

**Conclusions:**

The prevalence of polycythemia was lower compared to other similar studies done in similar settings.

## INTRODUCTION

Chronic Obstructive Pulmonary Disease is characterized by a chronic inflammatory response of the lungs to noxious particles.^1^ Polycythemia refers to an abnormal rise of Hemoglobin (Hb) and/or hematocrit (Hct) in peripheral blood which includes increased haemoglobin: greater than 16.5 g/dl in men or greater than 16.0 g/dL in women and Increased hematocrit: >49% for men and >48% for women.^2^ Despite anaemia being a common morbidity in COPD, severe COPD patients do experience secondary polycythemia which signifies the compensatory physiologic response to hypoxia.^3^ Polycythemia contributes to the development of cor pulmonale and pulmonary hypertension, which are linked to poor prognosis.^4^ Men, current smoking, impaired carbon monoxide diffusing capacity, severe hypoxemia, and high altitude living are risk factors associated with an increased risk for secondary polycythemia.^5^

Contrary to anaemia, polycythemia has been linked to decreased mortality and fewer hospitalizations among people with COPD who are administered long-term oxygen therapy (LTOT);^6^ however, the prevalence of polycythemia has not been thoroughly studied in a situation analogous to this.

This study aimed to find out the prevalence of polycythemia among patients with chronic obstructive pulmonary disease admitted to the department of medicine in a tertiary care centre.

## METHODS

A descriptive cross-sectional study was conducted at Gandaki Medical College and Teaching Hospital, (GMCTH) from 15 September 2022 to 2 December 2022. Ethical approval was taken from the Institutional Review Committee of GMCTH (Reference number: 153/079/080). The study included all the patients diagnosed with COPD and categorized according to the GOLD criteria 2022 and admitted to the medicine department whereas incomplete hospital records/missing data, patients with a history of malignancy or haematological disorder, systematic or autoimmune disorder, thyroid disease, liver cirrhosis, heart failure (ejection fraction <55%), history of gastrointestinal or other haemorrhages, renal failure (serum creatinine >1 mg/dl) and blood transfusion in the last 4 months were excluded. A convenience sampling method was used.

The sample size was calculated by using the formula:


$$\begin{array}{ll} \text{n} & ={\text{Z}}^{2}\times \frac{\text{p}\times \text{q}}{{\text{e}}^{\text{2}}}\\ & =1.96^{2}\times \frac{0.50\times 0.50}{0.08^{2}}\\ & =150 \end{array}$$

Where,

n = minimum required sample size
Z = 1.96 at 95% Confidence Interval (CI)
p = prevalence taken as 50% for maximum sample size calculation
q = 1-p
e = margin of error, 8%

The calculated sample size was 150. After adding 10% missing data the sample size is 167. However, a total of 185 subjects were taken for the study. The attending physicians made the diagnosis of COPD based on the clinical history, examination findings, and radiological and epidemiological manifestations. COPD severity was determined based on a global initiative for chronic obstructive lung disease (GOLD) guidelines.^5^

The necessary data were collected from the hospital records after taking permission from the Department of Medicine of the same hospital. Data were entered and analyzed using Microsoft Excel 2016. Point estimate and 95% CI were calculated.

## RESULTS

**Figure 1:**
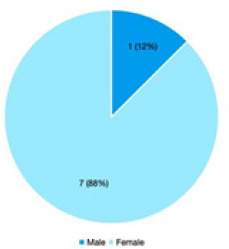
Gender-wise distribution (n = 8).

The prevalence of polycythemia was 8 (4.32%) (1.397.25, 95% CI). In our study among the patients with polycythemia, the prevalence of polycythemia was 7 (87.5%) in female and 1 (12.5%) in male (Figure 1).

Among the 8 patients, 7 were from the age group 70 and above and 1 was from the age group 50-59 (Table 1).

**Table 1:** Age-wise distribution (n= 8).

Age (years)	n (%)
<50	-
50-59	1 (12.50)
60-69	-
>70	7 (87.50)

**Figure 2:**
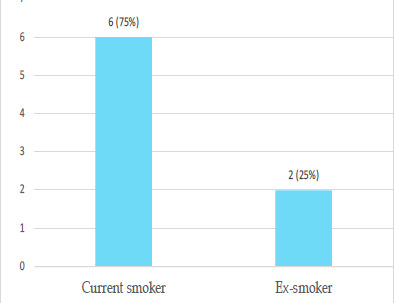
Smoking status (n= 8).

The majority of the patients with polycythemia were the residents of the Hilly region of Nepal 6 (75%) whereas 2 (25%) were from Terai and none were from the Himalayan region. The mean age of years since diagnosis of COPD in a patient with polycythemia was 12.875±2.295. The mean RBC count was 6.371 ±0.58. The mean hematocrit concentration, haemoglobin concentration, and PCV were 54.037±3.821, 17.51±1.105, and 51.407±3.915 respectively. Among the patients with Polycythemia 6 (75%) were current smokers and 1 (12.5%) was an ex-smoker with a mean pack year of 25.25±3.807 (Figure 2). There were 2 (25%) patients in the study with polycythemia who were under LTOT.

## DISCUSSION

The prevalence of polycythemia in our study was 8 (4.32%) which is lower as compared to a similar study conducted in Nepal,^7^ whereas prevalence is higher than in the study done in China.^8^ This difference might be due to different races and altitudes as reported by another study.^5^ Despite the presence of different criteria to define polycythemia, the prevalence of polycythemia in this study, which was 4.324%, does not match previous estimates of 5.9-18.1%.^9,10^

Although hypoxia stimulation is believed to cause quantitative changes while inflammation causes qualitative changes, the effects of both on the hematopoietic system were balanced.^11^ In other words, in severe COPD, more severe inflammation leads to a clear EPO resistance or haematological malfunction.^12^ Its low prevalence may, at least in part, be attributed to the frequent need for LTOT in people with severe COPD. However, in our study, only 2 (25%) patients with polycythemia were under LTOT. The low prevalence in our study might be due to the difference in the severity of the patient population included in our study. As the GOLD stage was not classified in any of the patients with polycythemia in our study group, we are not able to predict the exact severity status of our study population so further study with samples adequately classified based on COPD severity will provide additional ideas about it.

The prevalence of polycythemia is higher in current smokers 6 (75%) compared to Ex-smoker 2 (25%) which is similar to the study done in the United States.^5^ The prevalence of polycythemia is seen higher in patients without long-term oxygen therapy 6(75%) as compared to those who are under long-term oxygen therapy 2 (25%). In contrast, 8.4% of more than 2500 French patients with severe COPD using LTOT had a hematocrit of more than 55 %.^13^

In our study among the patient with polycythemia, the prevalence of polycythemia was higher in Female 7 (87.5%) vs. Males 1 (12.5%) which is in contrast to the study done in the United States which might be due to the higher prevalence of COPD among females in Nepal.^5,7^ Living at altitude and being a current smoker were related to higher rates of polycythemia, which was also linked to decreased resting but not exertional oxygen saturation. As a result, nearly one-fifth of people with secondary polycythemia may have unrecognized risk factors such as awake or nocturnal hypoxemia. However, not all of the individuals with polycythemia in this study had one of the conventional risk factors identified.^14^ Therefore, the presence of polycythemia without clearly defined risk factors should trigger further research, such as ambulatory or nocturnal oximetry, which may identify clinically significant hypoxemia necessitating intervention that could improve quality of life, increase exercise tolerance, or prevent cognitive impairment.^15,16^

Our study is constrained by the relatively small sample size; a bigger sample size is needed in future research to more accurately estimate the prevalence of polycythemia in COPD patients. Only Hb, hematocrit, and PCV values were considered in our study to rule out polycythemia because of the lack of facility in our settings; however, it would be preferable to assess serum erythropoietin and red cell mass as well. Variations in Hb concentration throughout follow-up were not recorded, and this study neglected the history of disease exacerbation and the method of hospital admission, both of which may have an effect on erythropoiesis and Hb concentration. Future research should examine these possible relationships.

The lack of classification according to GOLD criteria for disease presentation was a drawback which limited our study to identify variations of polycythemia based on COPD severity.

## CONCLUSIONS

The prevalence of polycythemia in COPD in our setting was lower compared to other similar studies done in similar settings.
